# Undifferentiated large cell/rhabdoid carcinoma presenting in the intestines of patients with concurrent or recent non-small cell lung cancer (NSCLC): clinicopathologic and molecular analysis of 14 cases indicates an unusual pattern of dedifferentiated metastases

**DOI:** 10.1007/s00428-021-03032-6

**Published:** 2021-01-27

**Authors:** Abbas Agaimy, Ondrej Daum, Michal Michal, Mona W. Schmidt, Robert Stoehr, Arndt Hartmann, Gregory Y. Lauwers

**Affiliations:** 1 Institute of Pathology, Friedrich-Alexander-University Erlangen-Nürnberg, University Hospital Erlangen, Erlangen, Germany; 2 Department of Pathology, Faculty of Medicine in Pilsen, Charles University, Prague, Czech Republic; 3 Department of General, Visceral and Transplantation Surgery, University of Heidelberg, Heidelberg, Germany; 4 Department of Pathology, H. Lee Moffitt Cancer Center & Research Institute and Departments of Pathology and Cell Biology and Oncologic Sciences, University of South Florida, Tampa, FL USA

**Keywords:** Anaplastic carcinoma, Rhabdoid carcinoma, Undifferentiated carcinoma, NSCLC, Large cell carcinoma, Gastrointestinal, Intestine, Metastasis, SWI/SNF, SMARCA4

## Abstract

Undifferentiated carcinoma metastatic to the bowel is uncommon in surgical pathology practice and might be confused with primary gastrointestinal carcinoma, melanoma, lymphoma, and others. We present 14 cases of uni- (*n* = 9) or multifocal (*n* = 5) undifferentiated large cell/rhabdoid carcinoma presenting in the bowel of patients with concurrent (*n* = 9) or recent (diagnosed 1 to 25 months earlier; median, 4) non-small cell lung cancer (NSCLC). Patients were 6 females and 8 males, aged 52 to 85 years. Primary NSCLC was verified histologically in 10 cases and by imaging in 4. The undifferentiated histology was present in the lung biopsy in 4/10 patients (as sole pattern in 3 and combined with adenocarcinoma in 1) and was limited to the intestinal metastases in the remainder. PDL1 was strongly expressed in 7/9 cases (CPS: 41 to 100). Loss of at least one SWI/SNF subunit was detected in 7/13 cases (54%). SMARCA2 loss (*n* = 6) was most frequent and was combined with SMARCA4 loss in one case. PBRM1 loss was observed in one tumor. Successful molecular testing of 11 cases revealed *BRAF* mutations in 4 (3 were non-V600E variants), *KRAS* mutations in 3, and wildtype in 4. None had *EGFR* mutations. Analysis of 4 paired samples revealed concordant *KRAS* (2) and *BRAF* (1) mutations or wildtype (1). Our study indicates that undifferentiated carcinoma within the intestines of patients with concurrent/recent NSCLC represents dedifferentiated metastasis from the NSCLC. Recognition of this unusual presentation is cardinal to avoid misdiagnosis with inappropriate therapeutic and prognostic implications.

## Introduction

Primary undifferentiated and rhabdoid carcinoma of the small bowel is rare [1]. Evidence that the neoplasm originates from preexisting adenomatous epithelial or intraepithelial neoplasia and/or presence of a conventional (intestinal) differentiated tumor component is helpful in confirming a primary cancer and excluding a metastasis [1]. Since its first description as “pleomorphic (giant cell) carcinoma of the intestine” by Bak and Teglbjaerg in 1989 [2], a total of 39 cases of undifferentiated rhabdoid carcinoma of the intestine have been reported in 2014 (reviewed in ref. [3]). In a recent follow-up study on 13 new cases, our group identified frequent loss/inactivation of different components of the Switch-sucrose non-fermentable (SWI/SNF) chromatin remodeling complex in most cases [4]. Furthermore, 25% of cases were multifocal within the same bowel segment or involving different intestinal loci [3, 4]. The frequent multifocal presentation of these cases prompted us to check carefully the clinical records to exclude the possibility of metastasis from another primary. We retrospectively identified one case with a clinical diagnosis of non-small cell lung cancer (NSCLC) based on characteristic imaging findings which lead to its exclusion from that series [4]. This observation prompted us to study this unusual presentation of NSCLC to gain insight in the clinicopathological and genetic characteristics of this subset of neoplasms and at the same time to alert pathologists to this potentially misleading and likely under-recognized presentation of metastatic NSCLC.

## Material and methods

The cases presented herein were identified in the consultation files of the authors. Remarkably, 10 cases were seen by one of the authors (A.A.) within a 4-year period (2017–2020) in consultation. None was previously reported. The samples were used in accordance with ethical guidelines for the use of retrospective tissue samples provided by the local ethics committee of the Friedrich-Alexander University Erlangen-Nuremberg (ethics committee statements 24.01.2005 and 18.01.2012). The tumor specimens were fixed in buffered formalin and embedded for routine histological examination. All cases were tested for expression of five SWI/SNF complex subunits which we have established in our laboratory for the assessment of undifferentiated malignancies. These are SMARCB1, SMARCA2, SMARCA4, PBRM1, and ARID1A and for the mismatch repair (MMR) proteins MLH1, MSH2, MSH6, and PMS2 by immunohistochemistry. Other immunophenotypic markers were evaluated based on the specific characteristics of each case and the differential diagnosis raised (Table 2). Immunohistochemical stains were performed on freshly cut 3-μm paraffin sections using a fully automated slide preparation system “Benchmark XT System” (Ventana Medical Systems Inc, 1910 Innovation Park Drive, Tucson, AZ, USA) and the following antibodies: pancytokeratin (clone AE1/AE3, 1:40, Zytomed), epithelial membrane antigen (EMA, clone E29, 1:200, Dako), vimentin (V9, 1:100, Dako), desmin (clone D33, 1:250, Dako), protein S-100 (polyclonal, 1:2500, Dako), SOX10 (polyclonal, 1:25, DCS), CD117 (anti-Human c-kit proto-oncogene product, polyclonal, 1:200, Dako), ERG (EPR3864, prediluted, Ventana), CD30 (clone HS-4, 1:20, Immunotech), ALK (D5F3, D5F3, 1:100, Cell Signaling), CK5 (clone XM26, 1: 50, Zytomed), CK7 (OV-TL, 1:1000, BioGenex), CK20 (KS20.8, 1:50, Dako), CDX2 (clone CX294, 1:30, Dako), p63 (4A4, 1:100, Zytomed), p40 (polyclonal, 1:100, Zytomed), TTF1 (8F7G3/1, 1: 500, Zytomed), NapsinA (MRQ-60, ready-to-use, Medac), SMARCB1/INI1 (clone MRQ-27, 1:50, Zytomed Systems, Berlin), SMARCA2 (polyclonal antibody, 1:100, Atlas Antibodies AB, Stockholm, Sweden), SMARCA4 (clone EPNCIR111A, 1:100, Abcam, Cambridge, UK), ARID1A (rabbit polyclonal antibody, ab97995, 1:100; Abcam), PBRM1 (clone CL0331; 1:50; Atlas Antibodies AB), MLH1 (clone ES05, 1:50, Dako), PMS2 (clone EP51, 1:40, Dako), MSH2 (clone G2-19-1129, prediluted, Ventana), MSH6 (clone MSH6, 1:300, BD Pharmingen), and PD-L1 (clone 28-8, 1:200, Abcam), according to the manufacturer’s instructions. Assessment of the SWI/SNF and MMR markers was done the same way as reported previously [4], i.e., only unequivocal clean absent staining in the nuclei of viable tumor tissue (away from necrotic areas) was considered “deficient or lost.” As a control, the presence of homogeneous strong nuclear staining of stromal fibroblasts, inflammatory cells, vascular endothelial cells, or normal epithelial cells in the background was a prerequisite for assessable staining in the tumor. “Reduced expression” was assigned if viable tumor cells displayed homogenous very weak but still recognizable staining as opposed to stronger staining in normal cells in the background.

### Molecular testing

Except for three cases (two with available primary tumor tissue and one with available material from a mediastinal lymph node metastasis), only material from each of the intestinal metastasis was available for analysis. For case 6, detailed external molecular report of the primary NSCLC was available for comparison with the intestinal metastasis. Tumor DNA was isolated after manual microdissection of highlighted tumor area. Amplicon-based massive parallel sequencing was performed using a commercial 15 gene panel, the TruSight Tumor 15 (TST15) panel, Illumina, San Diego, USA, and a MiSeq system according to the manufacturer’s instructions (Illumina). The 15 gene panel is focused on the detection of hot-spot mutations within the coding regions of 15 genes (*AKT1*, *BRAF*, *EGFR*, *ERBB2*, *FOXL2*, *GNA11*, *GNAQ*, *KIT*, *KRAS*, *MET*, *NRAS*, *PDGFRA*, *PIK3CA*, *RET*, *TP53*) frequently altered by mutations in solid tumors. Raw sequencing data was automatically aligned to the human genome (hg19), and the reported variants were annotated using Variant Studio 3.0 (Illumina). Notably, the NGS panel used encompasses mutations frequently seen in undifferentiated melanoma (*BRAFV600E*, *NRAS*, and *KIT*) and also all relevant coding exons of *KIT* and *PDGFRA* expected to be mutated in gastrointestinal stromal tumor (GIST).

## Results

### Critical reevaluation of the cohort

After critical review of the clinicopathological and molecular features of 16 cases with a diagnosis of NSCLC metastatic to the gastrointestinal tract, two cases have been excluded to maintain uniformity of this series. The first patient had a TTF1-positive tubular adenocarcinoma of the lung metastatic to the terminal ileum. The metastasis was histologically identical to his primary tumor (uniformly tubular and TTF1-positive). His adenocarcinoma was HepPar1-positive and showed loss of SMARCA2 and PBRM1 (data not shown). The second patient had a biopsy-proven lung adenocarcinoma followed by undifferentiated large cell metastasis in the intestine. The metastasis was pankeratin- and CK7-positive and lacked any other differentiation markers including negativity with five melanocytic markers. Molecular testing revealed however an *NRAS* p.Gln61His mutation. Given the rarity of this mutation in NSCLC and its frequency in undifferentiated and dedifferentiated melanoma, we preferred to exclude this case, although in our experience with > 85 undifferentiated melanomas, CK7 expression and association with lung adenocarcinoma is very unusual and has not been encountered [5, 6]. The remaining 14 cases fulfilled the criteria for inclusion in further analysis.

### Clinical features

Fourteen patients had a clinical diagnosis of intestinal metastases from lung cancer (Table 1). Six patients were females and 8 were males ranging in age from 52 to 85 years (median, 60). The intestinal metastases were either unifocal (*n* = 9) or multifocal (*n* = 5). The metastases and the primary NSCLC were synchronous in 9 cases and metachronous in 5 (presented 1, 4, 4, 8, and 25 months after diagnosis of NSCLC). The median interval between the NSCLC and subsequent intestinal metastasis was 4 months. Eleven patients had small bowel involvement only: jejunum (*n* = 5), jejunum + duodenum (*n* = 1), jejuno-ileal junction (*n* = 1), and unspecified (*n* = 4). Two patients had colonic metastases only (one unifocal and one multifocal). One patient with small bowel metastasis had concurrent other gastrointestinal metastases in the esophagus and the stomach. Case 6 (the one with the longest interval between primary tumor and bowel metastasis) presented with acute abdomen due to perforated metastasis 25 months after his NSCLC. He present with recurrent metastasis near the small bowel anastomosis 15 months later which was resected again. This patient, who has cerebral metastasis since first diagnosis of his NSCLC, is currently alive with controlled disease under immune checkpoint therapy (45 months after initial diagnosis).

**Table 1:** Clinicopathological features of patients with undifferentiated gastrointestinal metastases from NSCLC (*n* = 14)

No.	Age/gender	Site of MTS	Size cm	Time to MTS	Other organ MTS	Histology of MTS	Submitted diagnosis	Lung findings
1	63 M	Jejunum	NA	Syn	Oral cavity	Undifferentiated rhabdoid large cells	Unclassified large cell malignancy	14.5 cm peripheral LCC, invading pleura, pericardium & diaphragm (resected from right lung), 3 cm G1 adenocarcinoma in same specimen
2	59 F	Small bowel	13	Syn	NA	Undifferentiated rhabdoid large cells	Intestinal sarcomatoid carcinoma vs epithelioid sarcoma	Imaging consistent with lung cancer
3	66 F	Distal ascending colon, other colonic segment	NA	Syn	Pancreas, liver, kidney	Solid undifferentiated large cell pattern with rhabdoid & pleomorphic giant cells	Unclassified malignancy	Imaging consistent with lung cancer
4	76 M	Multiple (n=5) duodenal (near papilla) and jejunum	3	Meta – 8 months	NA	Solid undifferentiated large cell pattern	Unclassified malignancy	Biopsy-proven TTF1-positive adenocarcinoma
5	59 F	Small bowel segments (x2)	NA	Syn	NA	Solid undifferentiated large cell pattern with rhabdoid cells	Unclassified malignancy	Biopsy-proven (EBUS-TBNA) acinar-papillary (<2%) + large cell undifferentiated carcinoma
6	57 M	Jejunum	NA	Meta (25 months) 15 months later jejunal rec	Syn: LNs, brain, adrenal	Anaplastic rhabdoid	SWI/SNF-deficient rhabdoid carcinoma?	Biopsy-proven NSCLC not specified
7	58 F	Multiple – small bowel	NA	Syn	NA	Monomorphic cells, vesicular nuclei, prominent nucleoli, eosinophilic cytoplasm	NA	Biopsy-proven CK7, NapsinA, & TTF1-positive adenocarcinoma
8	52 F	Multiple ulcerated transmural jejunal tumors	NA	Syn	Mediastinal, abdominal & axillary nodes	Pleomorphic giant cell rhabdoid	NA	Imaging consistent with lung cancer
9	55 M	Jejuno-ileal transition	NA	Meta – 4 months	Not known	Solid poorly differentiated, focally dyscohesive, rarely rhabdoid	NA	Biopsy-proven G2 SCC
10	62 M	Jejunum	NA	Meta – 1 month	Left adrenal	Undifferentiated solid, focally of syncytial-like appearance	NA	Biopsy-proven solid poorly differentiated carcinoma (CK7+, CK5/6-)
11	80 F	Jejunum	NA	Syn	NA	Solid undifferentiated large cell pattern	Solid angiosarcoma	Biopsy-proven central LCC, p63+
12	77 M	Small bowel	NA	Syn	NA	Undifferentiated rhabdoid large cells	Poorly differentiated carcinoma?	Imaging consistent with cT4 lung cancer
13	58/M	Small bowel	2.8	Meta – 4 months	Esophagus,adrenal, bone, stomach, omentum (4 months)	Undifferentiated large cell + solid adenocarcinoma	in-house case	Biopsy-proven solid adenocarcinoma (CK7+, HepPar1+, TTF1-, p40-)
14	85 M	Cecum	NA	Syn	NA	Solid undifferentiated large cell pattern with rhabdoid cells	Unclassified malignancy	Biopsy-proven TTF1-negative adenocarcinoma

*F*, female; *LCC*, large cell carcinoma; *male*, male; *MTS*, metastasis; *Meta*, metachronous; *NA*, not available; *rec*, recurrence; *SCC*, squamous cell carcinoma; *Syn*, synchronous

All patients but one underwent surgical resection due to acute symptoms or mass effect. Six patients presented with additional organ involvement by metastatic disease including 3 with adrenal metastases. In 2 of these 6 patients with multiorgan disease, the intestinal metastases were multifocal.

### Pathological findings of the GI metastases

The differential diagnoses raised by the referring primary pathologists were available for 9 cases. Undifferentiated/unclassified large cell malignancy was most frequently suggested (*n* = 5), followed by sarcomatoid, poorly differentiated or SWI/SNF-deficient rhabdoid carcinoma of the bowel (*n* = 3), and angiosarcoma (*n* = 1). Overall, a diagnosis of metastatic NSCLC was not favored or rendered by the referring pathologist in any of the cases.

**Fig. 1:**
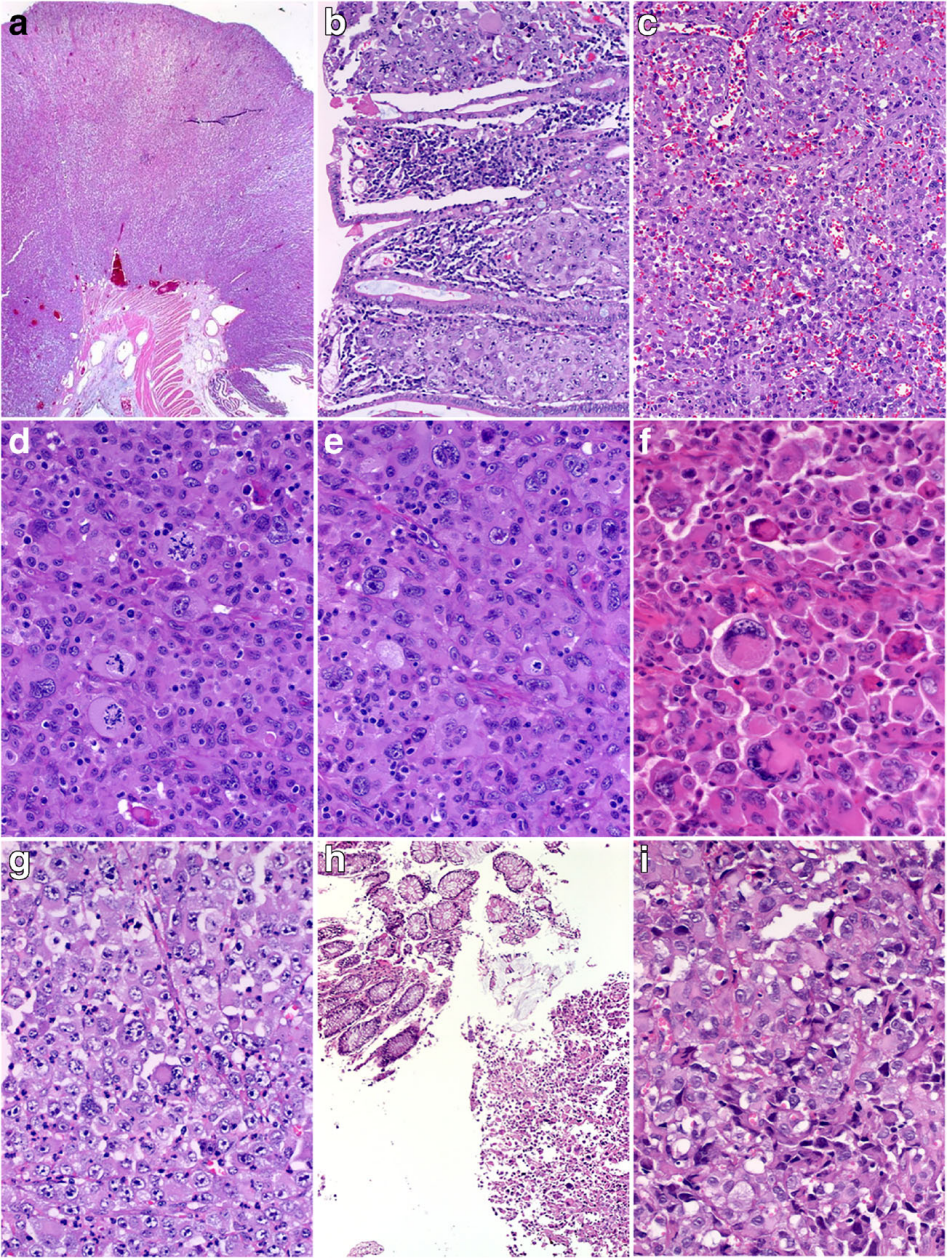
Representative examples of metastatic undifferentiated NSCLC in the GI tract. **a** Overview of ulcerated polypoid jejunal metastasis from undifferentiated NSCLC. **b** Extensive infiltration of the villous lamina propria mucosae is seen frequently at the periphery of ulceration. **c** Especially in the superficial parts, the prominent stromal vascularization and acantholytic pattern closely mimic angiosarcoma. **d** Prominent mitotic activity and karyorrhexis are seen. **e** Scattered binucleated Hodgkin- and Reed-Sternberg-like cells and admixed small lymphocytes may suggest Hodgkin lymphoma or anaplastic large cell lymphoma. **f** Prominent rhabdoid cell morphology indistinguishable from rhabdoid melanoma is frequently seen, at least focally. **g** Epithelioid large cell pattern with vesicular chromatin and prominent neutrophilic infiltration closely mimicking epithelioid inflammatory myofibroblastic sarcoma. **h** This SMARCA4/A2-deficient case presented with multiple colonic polyps that were biopsied. I: at high power, large anaplastic variably rhabdoid-looking cells are seen

All metastases presented as strikingly polypoid transmural masses with extensive superficial ulceration and diffuse infiltration of the surrounding mucosa, submucosa, and the muscle layer at the periphery of the mass (Fig. 1a, b). They were composed of diffuse sheets of large poorly cohesive cells with variably vesicular nuclei, prominent centrally located eosinophilic macronucleoli, and prominent rhabdoid morphology with significant mainly neutrophilic mixed inflammatory infiltrates in the background stroma (Fig. 1c–g). In the most superficial aspect of the tumor, prominent stromal vascularization and pseudoangiosarcomatous patterns were frequently observed (Fig. 1c). Mitotic figures including atypical forms and karyorrhexis were easily identified (Fig. 1d). Another frequent and characteristic feature seen in all cases, at least focally, was the presence of bi- or multinucleated neoplastic cells closely mimicking Hodgkin and Reed-Sternberg cells or recapitulating the convoluted blastic cells seen in anaplastic large cell lymphoma (Fig. 1e). In other areas, frankly rhabdoid large eosinophilic cells with enlarged peripherally displaced nuclei reminiscent of melanoma cells were evident (Fig. 1f, g). The sole tumor (in the cecum) that was only biopsied showed highly anaplastic cells bordered by normal colonic crypts (Fig. 1h, i). There was no evidence of adenocarcinomatous component and the overlying mucosa lacked intraepithelial neoplasia (Fig. 1h).

### Immunohistochemistry

**Fig. 2:**
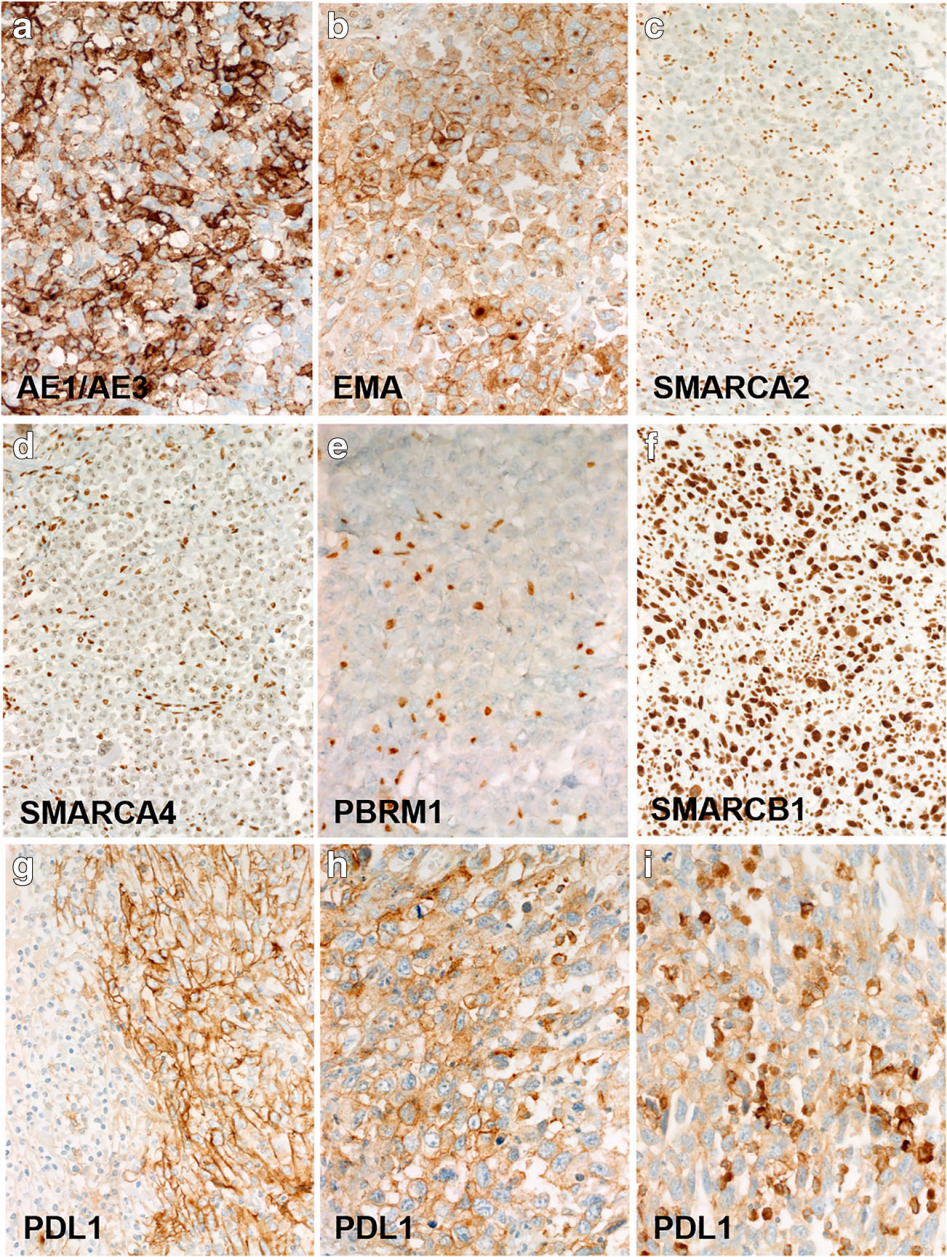
Representative examples of immunohistochemical findings in undifferentiated NSCLC metastatic to the GI tract. Variable expression of pankeratin (**a**) and/or EMA (**b**) was seen in all cases (not prominent Golgi-pattern in **b**). Loss of SMARCA2 was the most frequent SWI/SNF abnormality (**c**) and it was combined with loss of SMARCA4 (**d**) in one case. One case showed PBRM1 loss (**e**). SMARCB1 was intact in all cases (**f**). Of those cases tested for PDL1, all showed moderate to strong expression in the neoplastic cells (**g**, **h**). The immune cells varied from totally negative (**g**; left field) to strongly positive (**i**)

In line with their epithelial origin, immunohistochemical evaluation showed expression of pankeratin and/or EMA in all cases (Fig. 2a, b). TTF-1 (0/14) and NapsinA (0/8) were negative in all cases tested (no reserve slides or blocks were available for other cases). CK7 expression was observed in 5 of 12 cases tested. Only 2 of 13 cases tested for squamous cell markers showed limited weak reactivity for p63 and/or CK5. All other markers variably evaluated on a case-by-case basis and including endothelial, mesenchymal, melanocytic, hematolymphoid, and other lineage-specific markers were negative (Table 2). The most sensitive melanocytic markers S100 and SOX10 tested negative in all cases (0/12 and 0/14, respectively).

**Table 2:** Immunohistochemical and molecular features of undifferentiated gastrointestinal metastases from NSCLC (*n* = 14)

No.	Positive epithelial marker/s	Adenocarcinoma markers	Squamous markers	Other negative markers	PDL -1 tumor/immune cells	Lost SWI/SNF subunit	MMR status	TST15 gene status
1	EMA	TTF1 & NapsinA neg	CK5 neg	CD30, ALK, SATB2, synaptophysin, desmin, NUT, SALL4, HepPar1, ERG, AE1/AE3, OSCAR, CK34betaE12, CK18, CK7, S100, SOX10	NR	Retained	NR	*BRAF* p.Val600_Lys601de (AF: 50%)
2	EMA, AE1/AE3	TTF1 neg	CK5 & p40 neg	PAX8, p40, CK7, CK20, CDX2, CK5/6, chromogranin, CD34, CD117, desmin, ALK, ERG, S100	80%/ 1% (CPS: 81%)	SMARCA2	Retained	*BRAF* p.Asp594Tyr (AF: 63.5%), No *EML4/ALK* or *ROS1* fusions
3	AE1/AE3	TTF1 neg	p63 neg	CDX2, SATB2, PAX8, p63, CK7, CK20, EMA, HepPar-1, desmin, Ch-A, synaptophysin, CD45, ALK, ERG, SOX10, MelanA, HMB45	80%/ 10% (CPS: 90%)	SMARCA4 & SMARCA2	Retained	*BRAF* V600E AF: 42%
4	AE1/AE3	TTF1 neg	CK5 neg, p63+ (F)	CDX2, CK20, SATB2, PAX8, CK7, EMA, ALK, HepPar1, synaptophysin, Ch-A, CD45, CD117, DOG1, ERG, SOX10, MelanA, HMB45	40%/ 1% (CPS: 41%)	SMARCA2	Retained	WT
5	AE1/AE3	TTF1 & NapsinA neg	CK5 & p63 neg	CDX2, SATB2, PAX8, CK7, EMA, SOX10, ALK, HepPar1, ERG, S100, SOX10	0%/ 1% (CPS: 1%)	Retained	Retained	*KRAS*: p.Gly12Val (AF: 39%) *PIK3CA*: p.Glu545Lys (AF: 21%)
6	CK18, EMA	TTF1 & NapsinA neg	CK5 & p40 neg	AE1/AE3, CD34, CD45, desmin, DOG1, CK7, CD31, HepPar1, synaptophysin, Ch-A, ERG, S100, SOX10	30%/ 80% (CPS: 100)	SMARCA2	Retained	Same *KRAS* p.Gly12Arg in primary & metastasis (AF: 70%)
7	AE1/AE3, CK7	TTF1 neg	NA	CDX2, CK20, S100, SOX10, Melan-A	NR	NR	NR	No *ALK* or *ROS*1 fusions, no *BRAF* & *EGFR* mutations
8	AE1/AE3	TTF1 & NapsinA neg	CK5 & p40 neg	HepPar1, CAM5.2, CD30, CD117, desmin, ERG, SOX10, S100, HMB45	NR	Retained	Retained	NR
9	AE1/AE3, CAM5.2, EMA	TTF1 & NapsinA neg	CK5 & p40 neg	HepPar1, Ch-A, NSE, ERG, SOX10, S100, HMB45	NR	SMARCA2	NR	NR
10	AE1/AE3, CK7, CK20 (wk)	TTF1 & NapsinA neg	CK5 & p63 wk+	HepPar1, ERG, SOX10, S100, HMB45, Melan A	NR	SMARCA2	NR	NR
11	AE1/AE3, CK7	TTF1 neg	CK5, p40 & p63 neg	Desmin, CD34, DOG1, CK20, NUT, CD31 (ERG wk+), ALK, CD30, SOX10	95%/ 5% (CPS: 100%)	Retained	NR	Same *BRAF* p.Asp594Asn (c.1780G>A) in primary & metastasis (AF: 19%)
12	AE1/AE3, CK7, EMA	TTF1 & NapsinA neg	CK5 & p63 neg	pan-melanoma, ERG, synaptophysin, SATB2, DOG1, CDX2, CD34, CD45, NKX3.1, prostein, PSA, CK20, NUT, ALK, SOX10, S100, HMB45	95%/ 1% (CPS: 96%)	Retained	Retained	WT
13	AE1/AE3, CK7, HepPar1++	TTF1 & NapsinA neg	CK5 & p63 neg	CDX2, CK20, ALK, ERG, SOX10, S100	0%/ 0% (CPS: 0%)	PBRM1	Retained	primary & metastasis WT, no *EML4/ALK* or *ROS1* fusions
14	AE1/AE3, CK18	TTF1 neg	p40 neg	CDX2, CK20, SATB2, PAX8, CK7, EMA, ALK, HepPar1, synaptophysin, Ch-A, CD45, CD117, DOG1, ERG, SOX10, S100	100%/ 50% (CPS: 100%)	Retained	Retained	Same *KRAS* p.Gly12Val in mediastinal nodal (48%) & Cecal metastasis (72%)

*AF*, allele frequency; *CH-A*, chromogranin-A; *CPS*, combined positivity score; *F+*, focal positive; *i+*, isolated cells positive; *IHC*, immunohistochemistry; *neg*, negative; *NR*, no results due to poor tissue quality; *wk*, weak; *WT*, wildtype

One of 13 tumors (case 12) showed weak expression of ERG (justifying consideration of solid epithelioid angiosarcoma by the referring pathologist), but it was negative for CD31 and lacked the homogeneous ERG expression characteristic of angiosarcoma. HepPar1 was strongly expressed in one of 9 tumors. SWI/SNF immunohistochemistry showed loss of at least one subunit in 7 of the 13 (54%) assessable cases. Loss of SMARCA2 was the most frequent SWI/SNF abnormality detected (6/13) and was isolated in 5 cases, and combined with SMARCA4 in one case (Fig. 2c, d). PBRM1 was lost in one case (Fig. 2E). None was SMARCB1- or ARID1A-deficient (Fig. 2f). All 9 tumors tested for MMR expression status were proficient. Seven of 9 cases tested successfully for PDL1 revealed moderate to strong expression in 30–95% of the neoplastic cell area (Fig. 2g, h). The associated immune cells were variable positive in all but one case (Fig. 2i). The combined positivity score (CPS score) ranged from 41 to 100% in the positive cases. One patient was treated with immune checkpoint inhibition therapy (case 6). He remained alive with controlled disease under maintenance immune therapy at last follow-up (45 months).

### Primary tumor histology

**Fig. 3:**
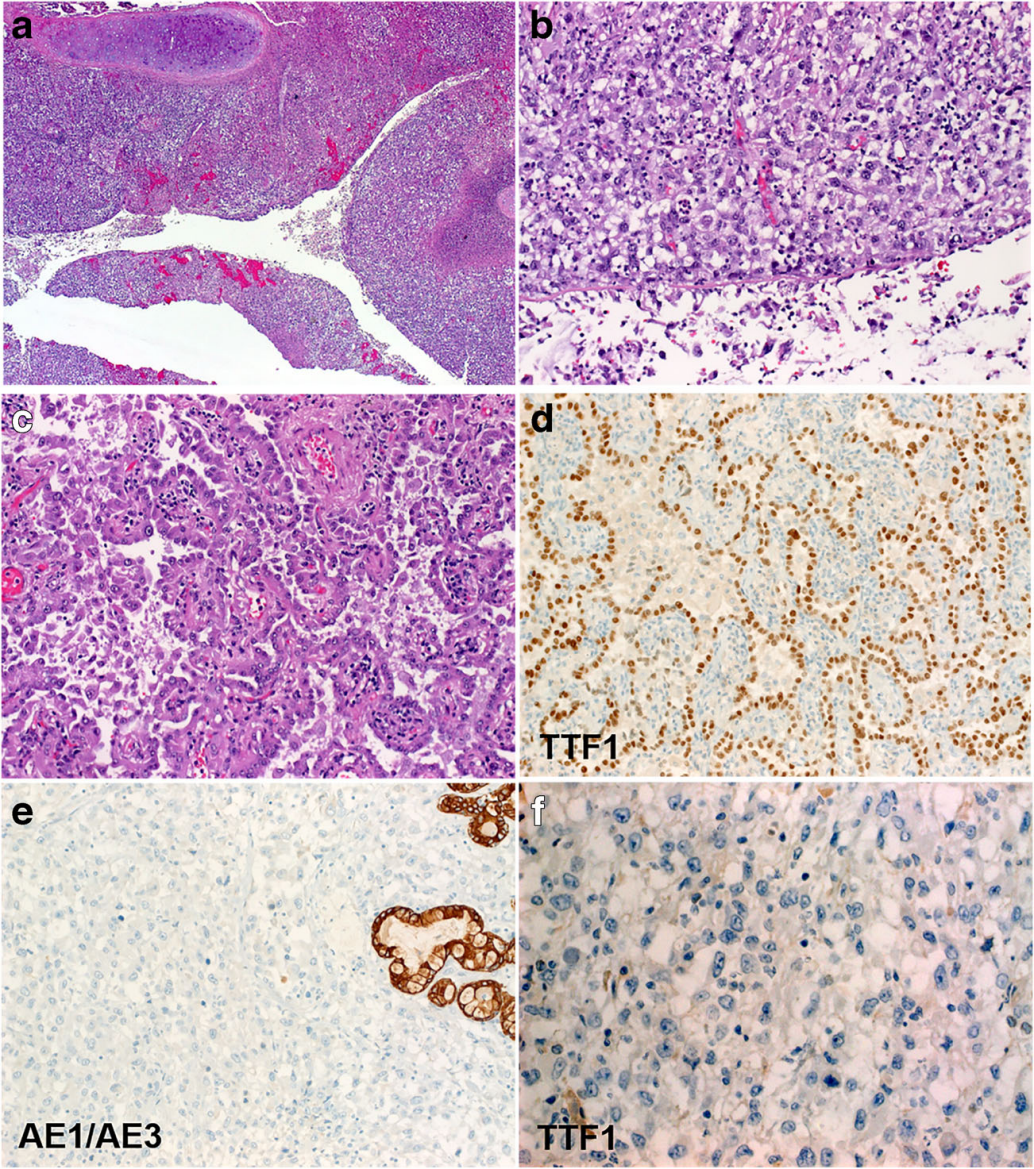
Representative images of the primary tumor in case 1. The resection showed predominantly undifferentiated carcinoma with extensive necrosis invading through the cartilage (**a**) and covered superficially by attenuated or exfoliated ciliated epithelium (**b**). Lepidic adenocarcinoma component was seen at the periphery (**c)** with retained TTF1-positivity (**d**). The large cell undifferentiated component showed loss of pankeratin AE1/AE3 (**e**; note staining in entrapped peribronchial glands) and TTF1 (**f**)

The histology of the primary tumor was evaluated on tissues obtained via either endobronchial or core biopsies in 9 cases and resection in one case (Fig. 3a, b). The undifferentiated histology was present in the lung biopsy in 4 of 10 patients (as sole pattern in 3 and combined with adenocarcinoma in 1) and was limited to the intestinal metastases in the remainder. Among the discordant cases, 3 primary tumors showed only an adenocarcinoma component on endobronchial biopsies and one was a grade 2 squamous cell carcinoma. In cases with combined adenocarcinoma-large cell undifferentiated carcinoma in the primary tumor, the metastasis was composed exclusively of the undifferentiated large cell component which has lost the TTF1 reactivity with variable loss of pancytokeratin (Fig. 3a–f). In one extensively sampled routine case (case 13) with a predominantly solid adenocarcinoma pattern in the primary tumor, the metastasis in the intestine was composed both of solid adenocarcinoma and undifferentiated large cell carcinoma.

### Mutational status

The molecular testing was successful in 8 metastases and in three pairs of both primary and metastasis (in one case, only needle biopsy from a mediastinal node was available for comparative testing). In case 6, only metastasis was available for molecular testing but the detailed molecular report of his primary NSCLC could be retrieved; we detected the same *KRAS* mutation (reported in his primary tumor) in the intestinal metastasis. Overall, 8 of 11 tumors showed mutations in genes frequently mutated in solid cancers including *BRAF* (4/11; 36%) and *KRAS* (3/11; 27%) mutations. Four tumors showed a wildtype status. *EGFR* mutations were absent in all cases. One *KRAS*-mutant case showed in addition a *PIK3CA* mutation*.* Remarkably, 3 of the four cases with *BRAF* mutations showed rare (non-V600E) variants involving codons 600–601 (1) and codon 594 (2). Only a single case had a *V600E* mutation. In case 12, in which both primary and metastasis were examined, the same *BRAF* mutation was detected in both samples, confirming a clonal origin. Case 14 had same *KRAS* mutation in the mediastinal node biopsy and the cecal metastasis and case 6 had same *KRAS* mutation in the primary NSCLC and the metastasis. No mutations were detected in the primary NSCLC or the metastasis in case 13. Taken together, molecular findings in 4 paired samples showed concordant positive (3) or negative (1) mutation status. Notably, none had mutations in hotspots regions of *KIT* or *PDGFRA* as would be expected in dedifferentiated GIST.

## Discussion

Although not uncommon at autopsy, clinically symptomatic secondary tumors of the gastrointestinal (GI) tract are relatively uncommon in surgical pathology practice; many representing contiguous invasion of the bowel in the setting of peritoneal carcinomatosis [7]. In one study comparing clinical and autopsy cases of GI metastases, melanoma (30%), ovarian (15%), bladder (11%), breast (8%), and lung (7%) cancers were the major tumor types encountered in surgical cases [7]. In contrast, the lung (with an incidence of 11 to 19%) outnumbered other entities in autopsy series followed by gynecologic malignancies (16%), breast (13%), and pancreas (8%) [7–10].

Metastatic NSCLCs presenting with intestinal symptoms are rare [11, 12]. Symptomatic small bowel metastases were diagnosed in 0.45% of patients with lung cancer referred to surgery [11]. No more than 58 cases of NSCLC presenting with symptomatic GI metastases have been published between 1961 and 2003 [13]. Likely due to their characteristic transmural aggressive growth pattern, NSCLC metastatic to the GI tract tends to present with serious life-threatening symptoms such as acute peritonitis, intestinal obstruction, perforation, and acute bleeding [13, 14]. The most common sites of small bowel metastases are the ileum and jejunum or both [13]. Synchronous metastases in other organs are present in most patients [13]. Finally, with a few exceptions, the development of GI metastasis is generally associated with poor outcome; most patients are dying within a year [7, 11, 15].

The interval between the diagnosis of primary lung tumor and the GI metastasis varied greatly (from synchronous to > 30 years) according to several autopsy series [7, 8]. However, clinically symptomatic metastatic cases are usually detected much earlier in the course of the disease, in the range of 0.5 to 24 months after the diagnosis of primary NSCLC and even preceding it in a few patients [9, 11].

Although large cell carcinoma metastases to the bowel seem overrepresented in previous studies and have been described in several single case reports [15–19], to our knowledge, the distinctive undifferentiated large cell rhabdoid morphology, the frequent histologic discrepancy between primary NSCLC and the metastasis, and the SWI/SNF expression status have not been studied in details before. This finding is likely significantly under-recognized and reflected by the frequency of such cases in our consultations, all sent with the question of independent second primary malignancy of the bowel, although the history of recent or concurrent NSCLC was provided. The confusion was further enhanced by the frequent discordance between the biopsy histology of primary tumors and their respective metastases. The case described by Sheikh et al. as metachronous malignant rhabdoid tumor of the ileum following lung adenocarcinoma likely represented the same phenomenon, in which the metastasis showed exclusively an undifferentiated rhabdoid cell morphology, distinct to the differentiated primary pulmonary adenocarcinoma [16]. Five of our cases presented the same histologic discordance. However, 2 tumors in our series showed combined adenocarcinoma (one in situ) and undifferentiated carcinoma. It is likely that the undifferentiated carcinoma component was missed on biopsies in the other cases.

The molecular findings of our series are also of interest. While rare *BRAF* mutation variants (involving codons 601 and 594) and *KRAS* mutations seem overrepresented in the 11 cases tested, it is remarkable that none showed *EGFR* mutations or neuroendocrine features.

As expected, given the anaplastic rhabdoid cell morphology, we detected frequent SWI/SNF protein loss in 54% of cases. Notably, loss of SMARCA2, occasionally combined with loss of SMARCA4, was the most common finding, detected in 46% of the cases. On the other hand, SMARCA4 was lost in only 8% of the cases. In an unselected series of NSCLC, loss of SMARCA4 and SMARCA2 was observed all together in 12% of adenocarcinomas (5.5% and 6.4%, respectively) compared to 6.9% (5.2% and 1.7%, respectively) of squamous cell carcinomas [20]. Of the few large cell carcinomas investigated in that study, 2/6 cases (33%) had a loss of SMARCA2 [20]. This finding is consistent with our current results showing that undifferentiated large cell morphology and SMARCA2 loss are significantly overrepresented among NSCLC metastases in the gastrointestinal tract. Notably, the excluded case of tubular adenocarcinoma and case 13 both expressed HepPar1 and had loss of PBRM1. Currently, no data is available on PBRM1 expression status in NSCLC. One recent study showed a frequency of *PBRM1* mutations of 3% in unselected NSCLC, but details on the immunohistochemical PBRM1 expression status were lacking [21]. In that study, *PBRM1* mutations in NSCLC were found as likely negative predictive biomarker for immune therapy but this needs further validation [21].

The major issues regarding these cases are as follows: (1) to differentiate them from primary rhabdoid carcinoma of the gut, (2) to distinguish them from undifferentiated metastatic melanoma, (3) to separate them from undifferentiated-looking other primary malignancies of the gut, and (4) to prove their pulmonary origin/ relation to the NSCLC.

Regarding the first point, primary rhabdoid intestinal carcinoma is very rare with <100 cases published since 1989 [3, 4]. This aggressive malignancy affects predominantly males at a mean age of 65 years [3]. The small bowel is uncommonly affected (26% of all cases) compared to the stomach and large bowel with 70% of the small bowel tumors being located in the jejunum; 13% of them being multifocal [3]. The pathogenesis of these multifocal cases is unclear. The affected patients had no evidence of extra-intestinal primary tumor on clinical and imaging examinations and/or at autopsy, thus suggesting either primary multifocal disease or discontinuous hematogenous spreading along the bowel wall itself. A differentiated component was observed in one third of cases [3, 4]. Thus, in cases without well-differentiated carcinoma component, rhabdoid carcinomas of the GI tract need to be distinguished from metastatic rhabdoid carcinoma of pulmonary or other origin. The SWI/SNF status represents another significant difference between primary rhabdoid intestinal cancer (where loss of SMARCB1, ARID1A, and SMARCA4 was observed collectively in 69% of cases [3, 4]), and metastatic undifferentiated NSCLC with no loss of SMARCB1/ARID1A and only rare SMARCA4 loss being observed in our series. These site-specific differences in the frequency of the SWI/SNF subunits involved in rhabdoid carcinomas remain obscure. One possible explanation might be the presence of different tissue-specific components and/or assembly variants of the SWI/SNF complex in the embryonic stem cells and progenitor cells across developmental stages in diverse organs [22].

Regarding the second point, metastatic melanoma may as well present as acute abdomen due to perforated or obstructive transmural intestinal metastasis in the bowel [5, 6]. Loss of immunomarkers and occasional aberrant expression of pancytokeratin in these cases may result in close mimicry to the cases described herein [5, 6, 23]. Accordingly, careful analysis of the clinical history is mandatory. Presence of unusual variants of *BRAF* mutations seen in 3 of our cases might be more in favor of metastatic NSCLC than melanoma, given the vanishing rarity of these variant mutations in dedifferentiated melanoma and, instead, the predominance of the classical *V600E* mutation in melanoma [5, 6, 23]. In a recent review study of >85 undifferentiated and dedifferentiated melanomas, these *BRAF* variants were never encountered in undifferentiated melanoma and *V600K* was the only non-V600E variant seen [6]. On the other hand, *NRAS* mutations slightly outnumbered *BRAF* mutations in undifferentiated metastatic melanoma with or without known primary [6]. This is why we preferred to remove the one case with detected *NRAS* mutation from the current series. Thus, and based on our experience with undifferentiated melanomas, none of the current cases qualifies for melanoma [6]. The potential value of actinic molecular signature in differentiating metastatic undifferentiated melanoma from rare *NRAS*-mutated NSCLC remains an issue of future studies [24–26].

Considering the third point, undifferentiated metastatic NSCLC should be distinguished from few other entities including in particular rhabdoid gastrointestinal stromal tumor (GIST), anaplastic large cell lymphoma (ALCL), and epithelioid inflammatory myofibroblastic sarcoma [27–29]. The detection of a conventional tumor component and/or immunoreactivity for CD117 and DOG1 are diagnostic of GIST. In difficult cases, *KIT* and *PDGFRA* mutation testing is a valuable adjunct. All of our 11 cases tested lacked mutations in these two genes, thus largely ruling out the possibility of dedifferentiated GIST. Anaplastic large cell lymphoma may closely mimic undifferentiated rhabdoid carcinoma and strongly express EMA [28]. Homogeneous expression of CD30 and cytotoxic markers and also of ALK in the majority of cases is diagnostic and rules out this consideration. Epithelioid inflammatory myofibroblastic sarcoma may closely mimic our cases as well, based on small intestinal location, the anaplastic epithelioid large cell morphology, and the prominent neutrophil-rich inflammatory reaction [29]. However, this rare entity has a strong predilection for young males (median age, 39) and usually lacks cytokeratin and EMA expression by immunohistochemistry [28]. Furthermore, it is defined by a distinctive nuclear membrane ALK reactivity pattern which is strongly associated with the presence of ALK-RANBP2 gene fusions [29].

Regarding the fourth and last point, proof of pulmonary origin in our current cases represents a complex issue. However, several features are strongly in favor of a pulmonary origin and can be considered confirmatory of a clonal origin with the primary NSCLC: (1) some tumors had a biopsy-proven primary undifferentiated large cell pulmonary carcinoma and histologically identical intestinal metastases, (2) others had combined adenocarcinoma-undifferentiated large cell carcinoma with their metastases showing exclusively undifferentiated large cell pattern, (3) the former makes it likely that cases with adenocarcinoma only in the EBUS biopsies possibly had an undifferentiated large cell carcinoma component that was missed in the biopsy, given the fact that most of the endobronchial and EBUS-TBNA biopsies are of significantly limited amount, (4) one tumor contained both adenocarcinoma and undifferentiated large cell carcinoma in the intestinal metastasis (this was the only in-house case with thorough sampling) making it possible that some undifferentiated metastases might have foci of adenocarcinoma missed on sampling the resection (only one block was available in most of consult cases), (5) detection of same *BRAF* (1 case) or *KRAS* (2 cases) mutation in the paired primary-metastasis represents a strong argument for clonal origin, (6) the distinctive SWI/SNF pattern that is different from primary rhabdoid intestinal cancer and more similar to large cell carcinoma of the lung, and (7) exclusion of other differential diagnoses pointed above. All these points are in line with undifferentiated metastases from the histologically proven or radiologically diagnosed NSCLC. Last but not the least, there is no plausible argumentation why should patients with NSCLC present in particular with intestinal malignancies that look uniformly large cell undifferentiated, given the exceptional rarity of undifferentiated rhabdoid small bowel cancer? Our study is limited by the unavailability of sufficient tissue from the primary tumor biopsies for comparative genotyping in most of the cases.

In summary, this series highlights a distinctive pattern of metastatic mainly synchronous NSCLC presenting as undifferentiated large cell carcinoma mimicking primary rhabdoid carcinoma of the intestine and other malignancies with predilection for small intestine (mainly jejunum). Recognition of this presentation is critical to avoid the misdiagnosis of these lesions as primary bowel cancer with ensuing inappropriate therapeutic and prognostic implications. The frequent expression of PDL1 and the very favorable clinical course of one patient who received maintenance immune therapy point to the clinical relevance of recognizing this unusual presentation of NSCLC which is otherwise considered a rapidly fatal disease. This is in particular relevant as PDL1 testing is not routinely used in gastrointestinal cancer. The relatively long follow-up (currently 45 months) of one of the patients with controlled disease highlights the benefit of immune therapy for this unusual presentation of metastatic NSCLC which has been previously considered rapidly fatal.
